# Associations between dual use of e-cigarettes and smoking cessation: A prospective study of smokers in England

**DOI:** 10.1016/j.addbeh.2019.106230

**Published:** 2020-04

**Authors:** Sarah E. Jackson, Lion Shahab, Robert West, Jamie Brown

**Affiliations:** Department of Behavioural Science and Health, University College London, London, UK

**Keywords:** E-cigarettes, Dual use, Quit attempts, Quit success, Smoking cessation, Nicotine replacement therapy

## Abstract

**Background:**

It has been claimed that use of e-cigarettes in combination with cigarettes outside of a quit attempt (‘dual use’) reduces quitting among smokers. This study aimed to assess whether dual e-cigarette users have lower smoking cessation rates than (i) exclusive cigarette smokers or (ii) dual users of nicotine replacement therapy (NRT) and cigarettes.

**Methods:**

Prospective cohort study of 1,498 smokers in England. The independent variable was dual use of e-cigarettes (*n* = 292), dual use of NRT (*n* = 117), or exclusive smoking (*n* = 1089), assessed at baseline. Outcomes were overall quit rate, past-year quit attempts, and success of quit attempts at 12-month follow-up. Baseline sociodemographic and smoking-related covariates were included.

**Results:**

Overall quit rates were not lower in dual e-cigarette users than exclusive smokers (OR = 1.31, 0.90–1.89). Dual users of e-cigarettes were more likely than exclusive smokers to make a quit attempt, but this difference was not significant after adjustment for covariates (OR = 1.27, 95%CI 0.95–1.69). Among those attempting to quit, success rates did not differ significantly. Dual users of e-cigarettes were less likely to make a quit attempt than dual users of NRT (OR = 0.61, 95%CI 0.38–0.98) but the success rate of quit attempts and overall quit rates did not differ significantly.

**Conclusions:**

In England, dual use of e-cigarettes is not associated with reduced overall quit rates compared with exclusive smoking or dual use of NRT. However, dual use of e-cigarettes is associated with a slightly higher quit attempt rate than exclusive smoking but lower than dual use of NRT.

## Highlights

• Smokers who use e-cigarettes are at least as likely to quit as exclusive smokers.
• They are slightly more likely to try to quit, but no more or less likely to succeed.
• Smokers who use NRT are the most likely to make a quit attempt.
• Smokers who use e-cigs are no less likely to quit than those who use NRT.

## 1 Background

On the basis of population-level trends and evidence from randomised controlled trials, there is increasing agreement that the use of e-cigarettes in a quit attempt aids smoking cessation (Beard et al., 2016, Hajek et al., 2019, Hartmann-Boyce et al., 2016, Zhu et al., 2017). However, there remains debate on how the use of e-cigarettes in combination with cigarettes outside of a quit attempt (‘dual use’) affects quitting, with claims being made that it severely undermines quitting (Aveyard et al., 2018, Glantz and Bareham, 2018, Kalkhoran and Glantz, 2016). This study directly addresses this question using longitudinal data to allow adjustment for critical variables that may be related to both uptake of e-cigarettes and cessation outcomes.

Each year, the majority of smokers in England (~70%) do not make a serious attempt to quit (West, Mohr, Proudfoot, & Brown, 2019). Approximately half report trying to cut down and smokers have to abstain temporarily from smoking every day because it is widely prohibited (West et al., 2019). For these reasons, many smokers report using e-cigarettes outside of quit attempts to reduce smoking (i.e., cutting down the number of cigarettes smoked each day) or use nicotine to temporarily abstain where smoking is prohibited (Patel et al., 2016, Simonavicius et al., 2017). Given that the most common reason for e-cigarette use is to quit smoking (Patel et al., 2016, Simonavicius et al., 2017), it is likely that many of these ‘dual users’ tried to stop smoking using an e-cigarette unsuccessfully but continued using e-cigarettes for these reasons, possibly with a residual desire to ultimately quit. Yet it has been claimed that this type of dual use might actually *reduce* quitting among smokers (de Andrade et al., 2013, Glantz and Bareham, 2018, McKee et al., 2014). Findings in the literature have been mixed, with the direction and magnitude of associations between dual use and cessation appearing to depend upon device type and frequency of use (Abrams et al., 2018, Biener and Hargraves, 2015, Brose et al., 2015, Hitchman et al., 2015, Manzoli et al., 2015, Villanti et al., 2018). On the basis of a *meta*-analysis of 20 studies, it has been proposed that aggregated across different frequencies of use and device types, dual use compared with exclusive cigarette use is associated with 28% lower odds of quitting smoking (Kalkhoran & Glantz, 2016). However, this *meta*-analysis has been criticised for selection bias and combining studies with heterogeneous designs, populations, measurements of exposure, and unmeasured confounders (Hajek et al., 2016, West et al., 2016). The focus on people who were smoking at baseline is misleading because it means those who tried e-cigarettes and quit were excluded, leaving only those who had tried e-cigarettes but carried on smoking, biasing the samples towards those less likely to be helped by e-cigarettes. In addition, they do not account for confounding factors, including that smokers who use e-cigarettes tend to be more dependent on nicotine and are more likely to have tried (and failed) to quit recently (Brown et al., 2014, Levy et al., 2017, Vardavas et al., 2015), which may reduce their odds of quitting. An observational design involving a relevant behavioural control and adjustment for important confounders could provide a clearer signal as to the association between overall dual use – as observed and aggregated across a representative sample of current smokers – and cessation outcomes.

A behavioural case-control design selects groups of people who are likely to have similar characteristics based on their behaviour. A relevant behavioural control in this case would be people who dual use cigarettes and medically licensed nicotine replacement products. There is evidence that dual use of nicotine replacement therapy (NRT) is associated with increased likelihood of making a quit attempt and achieving cessation (Beard et al., 2013, Moore et al., 2009, Silla et al., 2014). Dual users of NRT are likely to be more similar to smokers who dual use e-cigarettes than smokers who do not use e-cigarettes; indeed, a recent study reported comparable sociodemographic and smoking profiles of long-term users of NRT and long-term e-cigarette users (Jackson et al., 2019). This should reduce – but not eliminate – confounding by important psychological factors, such as motivation and dependence, and environmental factors, such as socioeconomic circumstances. Longitudinal data are required to provide appropriate adjustment for variables that may be related to both uptake of e-cigarettes and quitting behaviour (such as urges to smoke or motivation to quit). Finally, there is also a need to evaluate the impact of dual use of e-cigarettes on a complete set of outcomes (quit attempts, success of quit attempts, and overall quit rates).

This study aimed to assess prospective associations between dual use of e-cigarettes and tobacco and subsequent smoking cessation, while adjusting for key potential confounding factors measured at baseline. Specifically, we aimed to address the following research questions:

1. Among all current smokers, to what extent is dual use of e-cigarettes for any reason associated with later quit attempts and cessation, relative to dual use of NRT and exclusive smoking?
2. Among smokers who make a quit attempt between baseline and follow up, to what extent is dual use of e-cigarettes for any reason associated with odds of successful cessation, relative to dual use of NRT and exclusive smoking?
3. Are the same associations observed when the samples of dual users are restricted to those using e-cigarettes or NRT for smoking reduction? This research question was designed to account for dual users who use e-cigarettes or NRT for harm reduction likely being more interested in quitting than those who use these products for alternative reasons (e.g. in situations where smoking is not permitted). If dual use were to undermine quitting activity per se, this is the group that would arguably be most sensitive to detect any effect.
4. Are the same associations observed when the exposure (use of e-cigarettes, NRT, or neither) and outcomes are analysed cross-sectionally at follow-up, controlling for potential confounding by variables measured at baseline? This research question was designed to account for some of the factors that may make a smoker both more likely to become a dual e-cigarette user and less likely to make a quit attempt or quit successfully (e.g. level of addiction), and overcome the selection bias inherent in previous studies exploring dual use and quitting activity.

## 2 Materials and Methods

### 2.1 Design

The Smoking Toolkit Study is an ongoing monthly cross-sectional survey of representative samples of adults (≥16 years) in England. It is designed to provide insights into population-wide influences on smoking and cessation by monitoring trends on a range of variables relating to smoking (Fidler et al., 2011). It uses a form of random location sampling to select a new sample of approximately 1,700 adults aged ≥ 16 years each month. Participants complete a face‐to‐face computer‐assisted survey with a trained interviewer. Comparisons with national data indicate that key variables such as sociodemographics and smoking prevalence are nationally representative (Fidler et al., 2011).

### 2.2 Population

For the present study, we used aggregated data from respondents to the survey in the period from April 2015 (the first wave in which respondents were invited to participate in a 12-month follow-up) to February 2018 (the latest wave of the survey for which 12-month follow-up data were available).

**Fig. 1:**
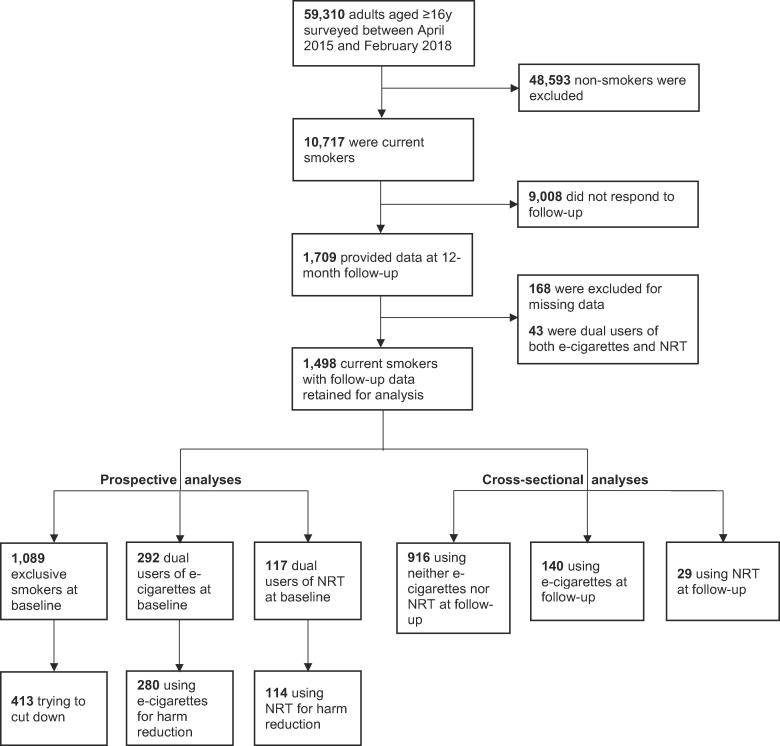
Summary of sample selection.

Our analytic sample was respondents who (i) reported smoking cigarettes (including hand-rolled) or other tobacco (e.g. pipe, cigar or shisha) daily or occasionally at the time of the baseline survey, (ii) provided complete data on current use of e-cigarettes and NRT and all covariates at baseline, and (iii) responded to the 12-month follow-up questionnaire. A flow diagram summarising the sample selection is shown in Fig. 1.

### 2.3 Measures

#### 2.3.1 Exposure

The exposure was dual use of e-cigarettes and tobacco. This was compared with two control groups: (i) dual use of NRT and tobacco, and (ii) exclusive smoking.

Three questions asked respondents to the baseline and 12-month follow-up questionnaires about use of e-cigarettes (or other vaping devices) or NRT:

1. Which, if any, of these are you currently using to help you cut down the amount you smoke? (This question is only asked if participants respond positively to the preceding question: ‘Are you currently trying to cut down on how much you smoke but not currently trying to stop?’)
2. Do you regularly use either of these in situations when you are not allowed to smoke?
3. Can I check, do you currently use either of the following at all for any reason?

For the primary analyses, a three-level exposure variable distinguished between smokers who reported use of e-cigarettes in response to any of these three questions at baseline (dual use of e-cigarettes, coded 1), those who reported use of NRT (dual use of NRT, coded 2), and those who neither reported use of e-cigarettes nor NRT (exclusive smoking, coded 3). Smokers who reported use of both e-cigarettes and NRT were excluded.

Subgroup analyses were performed on dual use for harm reduction purposes only, with smokers who reported use of e-cigarettes to help cut down the amount smoked coded 1, those who reported use of NRT to help cut down the amount smoked coded 2, and those who attempted to cut down but without the use of e-cigarettes or NRT for any reason coded 3.

In order to check how far results were influenced by confounding by variables associated with the uptake of e-cigarette use and quit attempts, a secondary analysis defined dual use according to responses to the three questions at 12-month follow-up: use of e-cigarettes by past-year smokers (coded 1), use of NRT by past-year smokers (coded 2), and use of neither e-cigarettes nor NRT by past-year smokers (coded 3). Past-year smokers who reported use of both e-cigarettes and NRT were excluded.

#### 2.3.2 Outcomes

Outcome variables, assessed at 12-month follow-up, were: (i) quit attempts in the entire sample of smokers, (ii) success of quit attempts among those who made a quit attempt, and (iii) the overall quit rate among smokers (i.e. the proportion of all smokers who quit).

Respondents to the 12-month follow-up were asked: “How many serious attempts to stop smoking have you made in the last 12 months? By serious attempt I mean you decided that you would try to make sure you never smoked again. Please include any attempt that you are currently making, and please include any successful attempt within the last 12 months.” Quit attempts were coded 1 for those who reported at least one quit attempt and 0 for those who reported no quit attempts in the last 12 months.

Respondents who indicated having made at least one quit attempt in the last 12 months were asked a follow-up question to evaluate the success of their most recent quit attempt: “How long did your most recent serious quit attempt last before you went back to smoking?” Quit success was coded 1 for those who answered “still not smoking” and 0 for those who answered “less than a day”, “less than a week”, “more than 1 week and up to a month”, “more than 1 month and up to 2 months”, “more than 2 months and up to 3 months”, “more than 3 months and up to 6 months”, or “more than 6 months and up to 12 months”.

Overall quits were coded 1 for respondents who reported having stopped smoking in the last 12 months and 0 for those who reported current smoking.

#### 2.3.3 Covariates

We included in our models a range of variables measured at baseline that are potentially associated with dual use of e-cigarettes and that may have an effect on quit attempts or abstinence.

Sociodemographic covariates included: age, sex, ethnicity (white vs. non-white), and social grade (an occupational index of socioeconomic position, categorised as ABC1, which includes managerial, administrative and professional and occupations, vs. C2DE, which includes semi‐routine and routine occupations, manual occupations, never workers and long‐term unemployed (Survey, 2007).

Smoking-related covariates were: number of cigarettes smoked per day, strength of urges to smoke (an indicator of cigarette addiction that closely predicts relapse in this population) (Fidler, Shahab, & West, 2011), motivation to stop smoking (Kotz, Brown, & West, 2013), and any serious attempt to quit smoking in the past year (yes/no). Analyses relating to the success of quit attempts also controlled for time since the quit attempt began and whether the quit attempt was abrupt or gradual, measured at 12-month follow-up.

Information on the year and quarter in which the survey was conducted were also included to take account of changes over the study period and seasonal variation in quit attempts.

### 2.4 Statistical analysis

The analysis plan was pre-registered on Open Science Framework (https://osf.io/tj5bh/). All analyses were conducted in SPSS v.24 on complete cases.

Simple associations between potential confounders and dual use of e-cigarettes were assessed with one-way independent analysis of variance (ANOVA) for continuous variables and chi-square tests for categorical variables.

For our primary analysis, we used multiple logistic regression models to analyse prospective associations between dual use of e-cigarettes at baseline and quit attempts, quit success, and overall quits at 12-month follow-up, relative to (i) dual use of NRT at baseline and (ii) exclusive smoking at baseline, adjusting for the above-mentioned covariates measured at baseline. We repeated these models limiting the sample to those who reported dual use of e-cigarettes for harm reduction (i.e. to cut down the amount smoked), dual use of NRT for harm reduction, or exclusive smokers who were trying to cut down to investigate the extent to which this produced different results. We also added an unplanned sensitivity analysis, on the suggestion of an anonymous reviewer, in which analyses of quit success were repeated with the sample of participants who had made a quit attempt restricted to those whose quit attempt had started more than one month prior (i.e., defining quit success as abstinence for at least one month at the time of the survey).

As a secondary analysis to check how far these results were influenced by confounding by variables associated with the uptake of e-cigarette use and quitting outcomes, we replicated our analyses on those who took up use of e-cigarettes or NRT between baseline and 12-month follow-up. Participants who were classified as exclusive smokers at baseline were categorised into three groups at 12-month follow-up: e-cigarette users, NRT users, and those who used neither e-cigarettes nor NRT. Multiple logistic regression models were used to analyse cross-sectional associations between use of e-cigarettes among past-year smokers at 12-month follow-up and quit attempts, quit success, and overall quits at 12-month follow-up, relative to (i) use of NRT among past-year smokers at 12-month follow-up and (ii) use of neither e-cigarettes nor NRT among past-year smokers at 12-month follow-up, adjusting for covariates measured at baseline.

Bayes factors (BF) were calculated to determine whether results were supportive of the alternative hypothesis (i.e. lower odds of quitting among dual users of e-cigarettes), the null hypothesis, or were insensitive. We used a conservative approach with half-normal distribution, the mode at 0 (no effect), and the standard deviation equal to the expected effect size (OR = 0.72 based on a previous *meta*-analysis of the association between e-cigarette use and smoking cessation (Kalkhoran & Glantz, 2016). BFs ≥ 3 can be interpreted as evidence for the alternative hypothesis (and against the null), BFs ≤ 1/3 as evidence for the null hypothesis, and BFs between 1/3 and 3 suggest the data are insensitive to distinguish the alternative hypothesis from the null (Dienes, 2014, Jeffreys, 1961).

## 3 Results

A total of 59,310 people responded to the baseline Smoking Toolkit Study survey between April 2015 and February 2018, of whom 10,717 (18.1%) were current smokers. Follow-up data were collected 12 months later from 1,709 baseline smokers. We excluded 168 participants with missing data and 43 who were dual users of both e-cigarettes and NRT, leaving a final sample for analysis of 1,498 men and women (Fig. 1). Supplementary Table 1 compares the analysed sample with baseline smokers who were excluded. The analysed sample overrepresented older and white smokers and underrepresented smokers from social grades C2DE, but the sex distribution was similar. They smoked more cigarettes per day and reported stronger urges to smoke than those who were excluded, and fewer reported high motivation to quit. The groups did not differ significantly on e-cigarette use but fewer smokers in the analysed sample than the excluded group reported using NRT.

**Table 1:** Sample characteristics at baseline.

		Whole sample (*n* = 1498)	Exclusive smokers (*n* = 1089)	Dual users of NRT (*n* = 117)	Dual users of e-cigarettes (*n* = 292)	*p*^1^
Age in years, % (*n*)						
	16–24	8.6 (1 2 9)	9.1 (99)	4.3 (5)	8.6 (25)	0.020
	25–34	10.8 (1 6 2)	10.4 (1 1 3)	8.5 (10)	13.4 (39)	–
	35–44	13.9 (2 0 8)	12.4 (1 3 5)	14.5 (17)	19.2 (56)	–
	45–54	20.0 (3 0 0)	20.1 (2 1 9)	24.8 (29)	17.8 (52)	–
	55–64	23.6 (3 5 3)	24.1 (2 6 2)	19.7 (23)	23.3 (68)	–
	≥65	23.1 (3 4 6)	24.0 (2 6 1)	28.2 (33)	17.8 (52)	–
Female sex, % (*n*)		47.9 (7 1 7)	48.0 (5 2 3)	54.7 (64)	44.5 (1 3 0)	0.173
White ethnicity, % (*n*)		94.1 (1410)	93.9 (1023)	94.9 (1 1 1)	94.5 (2 7 6)	0.874
Social grade C2DE, % (*n*)		50.6 (7 5 8)	51.3 (5 5 9)	51.3 (60)	47.6 (1 3 9)	0.521
Cigarettes per day, mean (SD)		12.59 (9.37)	12.47 (9.34)	12.85 (9.08)	12.96 (9.61)	0.694
Strength of urges (0–5), mean (SD)		2.01 (1.10)	1.93 (1.11)	2.27 (1.12)	2.22 (0.99)	<0.001
High motivation to quit, % (*n*)		12.8 (1 9 1)	9.5 (1 0 3)	30.8 (36)	17.8 (52)	<0.001
Attempted to quit in past 12 months, % (*n*)		27.5 (4 1 2)	19.5 (2 1 2)	53.0 (62)	47.3 (1 3 8)	<0.001
Dual use for harm reduction, % (*n*)		–	–	91.5 (1 0 7)	94.9 (2 7 7)	0.193

^1^*p* value for the association between each variable and group (dual use of e-cigarettes, dual use of NRT, and exclusive smoking).

At baseline, 292 (19.5%) smokers reported use of e-cigarettes (‘dual users of e-cigarettes’), 117 (7.8%) reported use of NRT (‘dual users of NRT’), and 1,089 (72.7%) did not report using either e-cigarettes or NRT (‘exclusive smokers’). Characteristics of these three groups at baseline are summarised in Table 1.

### 3.1 Prospective associations between dual use of e-cigarettes and quit attempts, quit success, and overall quit rate over 12-month follow-up

Prevalence of quit attempts over 12-month follow-up among participants who reported dual use of e-cigarettes at baseline was significantly higher than in exclusive smokers, but lower than in dual users of NRT (Table 2). After adjustment for covariates, the difference between dual users of e-cigarettes and exclusive smokers was not statistically significant, and the Bayes factor indicated the data provided moderate evidence for the null hypothesis compared with there being the expected negative association (BF = 0.12). The difference between dual users of e-cigarettes and dual users of NRT remained significant, but data were insensitive to detect the expected effect size (BF = 1.58).

**Table 2:** Prospective associations between dual use of e-cigarettes and tobacco at baseline and quit attempts, successful cessation among those who made a quit attempt, and overall quits at 12-month follow-up, adjusting for covariates measured at baseline.

		Prevalence, % (*n*)			OR [95% CI] *p*		OR_adj_ [95% CI]^1^*p*	
		Exclusive smokers (1)	Dual users of NRT and tobacco (2)	Dual users of e-cigarettes and tobacco (3)	(3) vs. (1)	(3) vs. (2)	(3) vs. (1)	(3) vs. (2)
Dual use for any reason, *n*		1089	117	292				
	Past-year quit attempts	34.5 (3 7 6)	61.5 (72)	47.9 (1 4 0)	1.75 [1.35–2.27]<0.001*	0.58 [0.37–0.89] 0.013*	1.27 [0.95–1.69] 0.108	0.61 [0.38–0.98] 0.039*
	Quit success^2^	35.0 (1 3 0)	25.7 (18)	29.5 (41)	0.78 [0.51–1.18] 0.238	1.21 [0.63–2.31] 0.567	0.89 [0.56–1.41] 0.621	1.16 [0.58–2.34] 0.678
	Overall quits	13.3 (1 4 5)	20.5 (24)	18.2 (53)	1.44 [1.02–2.04] 0.037*	0.86 [0.50–1.47] 0.581	1.31 [0.90–1.89] 0.161	0.91 [0.52–1.61] 0.752
Dual use for harm reduction, *n*		413^3^	114	280				
	Past-year quit attempts	44.6 (1 8 4)	60.5 (69)	47.9 (1 3 4)	1.14 [0.84–1.55] 0.392	0.60 [0.38–0.93] 0.023*	1.08 [0.78–2.84] 0.637	0.61 [0.37–0.98] 0.042*
	Quit success^2^	38.5 (70)	27.9 (19)	28.6 (38)	0.64 [0.40–1.04] 0.069	1.03 [0.54–1.98] 0.925	0.72 [0.42–1.23] 0.225	1.12 [0.55–2.31] 0.751
	Overall quits	18.6 (77)	21.1 (24)	17.5 (49)	0.93 [0.62–1.38] 0.702	0.80 [0.46–1.37] 0.411	0.98 [0.64–1.49] 0.916	0.83 [0.46–1.48] 0.520

^1^Adjusted for age, sex, ethnicity, social grade, number of cigarettes smoked per day, strength of urges to smoke, motivation to stop smoking, and past-year quit attempts at baseline, and year and quarter of survey. Models relating to the success of quit attempts also controlled for time since the quit attempt began and whether the quit attempt was abrupt or gradual, measured at 12-month follow-up.

^2^Among those who reported at least one quit attempt in the past 12 months.

^3^Exclusive smokers who reported trying to cut down.

* *p* < 0.05.

Prevalence of quit success over 12-month follow-up among smokers who reported having made at least one quit attempt did not differ significantly in dual users of e-cigarettes compared with either exclusive smokers or dual users of NRT (Table 2), although data were insensitive to exclude the possibility of a lower rate of success in dual users of e-cigarettes who made a quit attempt (BF = 0.84 and BF = 0.56, respectively). Excluding participants whose most recent quit attempt had started within the last month (*n* = 67) or who were unsure how long ago their quit attempt had started (*n* = 7) did not notably change the results (Supplementary Table 2).

The overall quit rate over 12-month follow-up was significantly higher among dual users of e-cigarettes than exclusive smokers (Table 2). After adjustment for covariates, the difference between dual users of e-cigarettes and exclusive smokers became non-significant, but still provided moderate evidence for the null hypothesis compared with the expected negative association (BF = 0.16). No significant difference in overall quits was observed between dual users of e-cigarettes and dual users of NRT, but data were insensitive to exclude the possibility of a lower quit rate in dual users of e-cigarettes (BF = 0.85).

Restricting the definition of dual use of e-cigarettes or NRT to those who reported using these products for harm reduction produced a very similar pattern of results (Table 2).

### 3.2 Cross-sectional associations between use of e-cigarettes among past-year smokers at 12-month follow-up and quit attempts, quit success, and overall quit rate

Among participants who were exclusive smokers at baseline, 140 (12.9%) reported current use of e-cigarettes at 12-month follow-up, 29 (2.7%) reported current use of NRT, and 916 (84.1%) reported using neither e-cigarettes nor NRT. Characteristics of these groups are summarised in Supplementary Table 3. Four (0.4%) reported current use of both e-cigarettes and NRT at follow-up, and were excluded from the following analyses.

**Table 3:** Cross-sectional associations at 12-month follow-up between use of e-cigarettes among past-year smokers and quit attempts, successful cessation among those who made a quit attempt, and overall quits, adjusting for covariates measured at baseline.

		Prevalence, % (*n*)			OR [95% CI] *p*		OR_adj_ [95% CI]^1^*p*	
		Neither e-cigarettes nor NRT (1)	Use of NRT (2)	Use of e-cigarettes (3)	(3) vs. (1)	(3) vs. (2)	(3) vs. (1)	(3) vs. (2)
Dual use for any reason, *n*		916	29	140				
	Past-year quit attempts	29.9 (2 7 4)	72.4 (21)	57.1 (80)	3.12 [2.17–4.49]<0.001*	0.51 [0.21–1.23] 0.132	2.83 [1.92–4.17]<0.001*	0.43 [0.17–1.08] 0.073
	Quit success^2^	33.5 (91)	40.0 (8)	39.7 (31)	1.31 [0.78–2.20] 0.305	0.99 [0.36–2.70] 0.983	1.43 [0.82–2.49] 0.214	0.85 [0.29–2.52] 0.768
	Overall quits	11.0 (1 0 1)	31.0 (9)	25.0 (35)	2.69 [1.74–4.16]<0.001*	0.74 [0.31–1.78] 0.501	2.82 [1.78–4.48]<0.001*	0.73 [0.29–1.84] 0.509

^1^Adjusted for age, sex, ethnicity, social grade, number of cigarettes smoked per day, strength of urges to smoke, motivation to stop smoking, and past-year quit attempts at baseline, and year and quarter of survey. Models relating to the success of quit attempts also controlled for time since the quit attempt began and whether the quit attempt was abrupt or gradual, measured at 12-month follow-up.

^2^Among those who reported at least one quit attempt in the past 12 months.

*p < 0.05.

Prevalence of quit attempts among past-year smokers who reported current use of e-cigarettes at 12-month follow-up was significantly higher than in those who used neither e-cigarettes nor NRT, but lower than in those who reported current use of NRT (Table 3). After adjustment for covariates, past-year smokers who currently used e-cigarettes had more than two times higher odds of having made at least one quit attempt in the past 12 months than those who used neither e-cigarettes nor NRT. Odds of having made a quit attempt were over 50% lower in e-cigarette users than NRT users, but this difference was not statistically significant and data were insensitive to exclude the possibility of a lower rate of quit attempts in e-cigarette users (BF = 1.11).

Prevalence of quit success among smokers who reported having made at least one quit attempt in the past 12 months was substantially higher among users of e-cigarettes or NRT than those who used neither (Table 3). After adjustment for covariates, the odds of success among those who made a quit attempt did not differ significantly between those who used e-cigarettes and those who used neither e-cigarettes nor NRT, and the data provided moderate evidence that the null hypothesis was more likely than the expected negative association (BF = 0.23). No significant difference was observed between users of e-cigarettes and users of NRT, but data were insensitive (BF = 0.99). Excluding participants whose most recent quit attempt had started within the last month (*n* = 59) or who were unsure how long ago their quit attempt had started (*n* = 7) saw differences between e-cigarette users and those who used either NRT or neither e-cigarettes nor NRT become more pronounced (Supplementary Table 4). Those who used e-cigarettes had higher odds than exclusive smokers of being successful in a quit attempt for at least one month (39.6% vs. 29.4%, OR_adj_ = 1.64, 95%CI 1.03–2.60). The small sample of NRT users who reported their most recent quit attempt starting at least one month prior to the survey (*n* = 40) meant we lacked statistical power to detect a significant difference between e-cigarette and NRT users (39.6% vs. 30.0%, OR_adj_ = 1.49, 95%CI 0.66–3.36).

The overall quit rates among smokers were substantially higher among users of e-cigarettes or NRT than those who used neither (Table 3). After adjustment for covariates, the odds of overall quitting were more than two times higher among those who used e-cigarettes than those who used neither e-cigarettes nor NRT. No significant difference was observed between users of e-cigarettes and users of NRT, but data were insensitive (BF = 1.05).

## 4 Discussion

Dual use of e-cigarettes was not associated with lower odds of quitting compared with exclusive smoking; quit attempt rates were higher and quit success rates among those who made a quit attempt did not differ significantly. Dual users of e-cigarettes had lower quit attempt rates than dual users of NRT.

Our findings conflict with the conclusions of a previous *meta*-analysis that dual use of e-cigarettes reduced the odds of smoking cessation by 28% (Kalkhoran & Glantz, 2016). Results of comparisons between dual use of e-cigarettes and dual use of NRT were not conclusive: the rate of quit attempts was lower among dual users of e-cigarettes but quit success and overall quits did not differ. However, the small number of participants in each of these groups meant data were insensitive. We had hypothesised that dual use of NRT may provide a more appropriate behavioural control for dual use of e-cigarettes than exclusive smoking, but the data insensitivity mean we are unable to draw firm conclusions regarding differences in quitting outcomes between these groups.

A key limitation of previous studies that have investigated the link between dual use of e-cigarettes and quitting behaviour is the lack of appropriate adjustment for confounding by factors that might be associated both with the uptake of e-cigarettes alongside tobacco smoking and the outcomes of interest. For example, dual e-cigarette users tend to smoke more heavily (Brown et al., 2014, Levy et al., 2017, Vardavas et al., 2015), indicating higher levels of addiction, and smokers who are more addicted tend to find it more difficult to quit (Borland et al., 2010, Fidler and West, 2011). In addition, by definition, restricting the sample to people who were currently smoking and using e-cigarettes means analyses were done on those whom dual use had failed to help or not yet helped to quit, without taking into account those whom dual use had already helped to quit. To address these issues, we analysed differences in quitting outcomes in relation to current use of e-cigarettes, NRT, or neither product among past-year smokers at 12-month follow-up, with adjustment for relevant covariates measured at baseline. By controlling for covariates one year prior to the measurement of exposure and outcome, this analysis was intended to account for variables that might make a smoker more or less likely both to take up e-cigarette use and to try to quit, overcoming problems of confounding by dependence and motivation to quit and widening the sample from treatment failures to also include new-uptake dual users. These results indicated that past-year smokers who used e-cigarettes were substantially more likely than those who used neither e-cigarettes nor NRT to have (i) made a serious attempt to quit and (ii) successfully quit smoking. One in four past-year smokers who used e-cigarettes were abstinent at 12-month follow-up, compared with one in ten who used neither e-cigarettes nor NRT.

Comparison of prospective and cross-sectional results indicates that adjustment for confounding factors measured prior to the exposure (dual use) and avoiding selection bias (i.e. restricting the sample to smokers whom e-cigarettes had not helped quit) substantially improves the apparent impact of e-cigarettes on quitting activity. In other words, when we include all smokers who use e-cigarettes in our analysis and not just those who have not yet quit successfully, e-cigarette use appears to be associated with increased odds of quitting. This finding is consistent with criticisms of previous efforts to examine the impact of dual use of e-cigarettes on smoking cessation (Hajek et al., 2016, West et al., 2016).

Strengths of this study include the prospective design, which made it possible to adjust statistically for potential confounding by variables that might influence uptake of e-cigarette use among smokers, and the inclusion of an appropriate behavioural control. However, there were several limitations. The optimal design for assessing the prospective association of dual use of e-cigarettes with quitting outcomes with appropriate adjustment for covariates would have been to use three waves of data, with covariates measured at baseline, dual use at the first follow-up, and quitting outcomes at the second follow-up. However, as this would require many years of data to accumulate, the current approach can provide insights now, albeit with the inability to distinguish between those who took up dual use of e-cigarettes or NRT between baseline and follow-up and those who started using these products after their quit attempt. Further research on a larger sample, using a three-wave longitudinal design, is needed to confirm or refute the present findings and provide further insight into differential effects of dual use of e-cigarettes and NRT on quitting. Other limitations include the reliance on recall of the last 12 months in the assessment of quit attempts and self-reported abstinence, which may have introduced bias. However, while a lack of biochemical verification would be a significant limitation in a randomised trial because smokers who receive active treatment may feel pressure to achieve abstinence, the social pressure and associated rate of misreporting in population surveys is generally low (West, Zatonski, Przewozniak, & Jarvis, 2007). Moreover, there is no reason in the present study to expect there to be differences in the extent of misreporting between groups. While we adjusted for a range of relevant sociodemographic and smoking characteristics, there may be residual confounding by unmeasured variables; for example, no data were available on health conditions that may influence smokers’ decisions around use of alternative nicotine and likelihood of trying to quit. In addition, no information was available on e-cigarette device type or frequency of use, which have been shown to be related to quitting outcomes in previous studies (Abrams et al., 2018, Biener and Hargraves, 2015, Brose et al., 2015, Hitchman et al., 2015, Manzoli et al., 2015, Villanti et al., 2018), so there is a need for further research into these aspects of dual use to provide a more nuanced understanding of its association with quitting. Finally, the definition of use of e-cigarettes or NRT for harm reduction was restricted to those who reported currently trying to cut down – but without currently trying to stop – and using nicotine products to help to reduce the amount they smoked. Another response option related to use in situations when they cannot smoke could also constitute use for harm reduction if it means users vape instead of leaving the area to smoke elsewhere. However, this question did not expressly exclude concurrent use for quitting. This may mean we excluded some participants from the subgroup analysis who were using these products for harm reduction, but given the small number of exclusions it is unlikely that this had a notable impact on our results.

## 5 Conclusions

Dual use of e-cigarettes does not appear to be associated with a lower rate of quit attempts or cessation, compared with exclusive smoking. Dual use of NRT compared with e-cigarettes was associated with greater odds of a quit attempt but data were inconclusive regarding differences between dual use of e-cigarettes and dual use of NRT on quit success and overall quit rates.

## Declaration of Competing Interest

JB has received unrestricted research funding to study smoking cessation from Pfizer, who manufacture smoking cessation medications. LS has received honoraria for talks, an unrestricted research grant and travel expenses to attend meetings and workshops from Pfizer, and has acted as paid reviewer for grant awarding bodies and as a paid consultant for health care companies. RW undertakes research and consultancy for and receives travel funds and hospitality from manufacturers of smoking cessation medications (Pfizer, GlaxoSmithKline and Johnson and Johnson). All authors declare no financial links with tobacco companies or e-cigarette manufacturers or their representatives.
