# Supplementary material for: Associations between dual use of e-cigarettes and smoking cessation: A prospective study of smokers in England
Source: Addict Behav. 2020 Apr;103:106230. doi: 10.1016/j.addbeh.2019.106230 (PMC6970222; doi:10.1016/j.addbeh.2019.106230)
Supplement: Supplementary data 2 [file mmc2.docx]

| **Supplementary Table 1.** Sample characteristics at baseline: comparison of analysed sample with excluded smokers | | | | |
| --- | --- | --- | --- | --- |
|  | | **Whole sample**  **(*n*=1498)** | **Excluded smokers**  **(*n*=9219)** | ***p*** |
| Age in years, % (*n*) | |  |  |  |
|  | 16-24 | 8.6 (129) | 20.5 (1893) | <0.001 |
|  | 25-34 | 10.8 (162) | 20.8 (1913) | - |
|  | 35-44 | 13.9 (208) | 16.3 (1499) | - |
|  | 45-54 | 20.0 (300) | 16.6 (1532) | - |
|  | 55-64 | 23.6 (353) | 13.5 (1243) | - |
|  | ≥65 | 23.1 (346) | 12.4 (1139) | - |
| Female sex, % (*n*) | | 47.9 (717) | 47.1 (4338) | 0.666 |
| White ethnicity, % (*n*) | | 94.1 (1410) | 88.7 (8134) | <0.001 |
| Social grade C2DE, % (*n*) | | 50.6 (758) | 60.4 (5572) | <0.001 |
| Cigarettes per day, mean (SD) | | 12.59 (9.37) | 11.05 (8.10) | <0.001 |
| Strength of urges (0-5), mean (SD) | | 2.01 (1.10) | 1.92 (1.13) | 0.004 |
| High motivation to quit, % (*n*) | | 12.8 (191) | 14.8 (1360) | 0.037 |
| Attempted to quit in past 12 months, % (*n*) | | 27.5 (412) | 28.2 (2514) | 0.576 |
| Using e-cigarette, % (*n*) | | 19.5 (292) | 19.5 (1795) | 0.984 |
| Using NRT, % (*n*) | | 7.8 (117) | 9.7 (891) | 0.023 |
| Note: There was some missing data in the group of excluded participants on the following variables: ethnicity, cigarettes per day, strength of urges to smoke, motivation to quit, past-year quit attempts. Valid percentages shown for ease of interpretation. | | | | |

| **Supplementary Table 2.** Prospective associations between dual use of e-cigarettes and tobacco at baseline and successful cessation (for at least one month) among those who made a quit attempt at 12-month follow-up, adjusting for covariates measured at baseline | | | | | | | | | | |
| --- | --- | --- | --- | --- | --- | --- | --- | --- | --- | --- |
|  | | **Prevalence, % (*n*)** | | |  | **OR [95% CI]**  ***p*** | |  | **OR_adj_ [95% CI]^1^**  ***p*** | |
|  | | **Exclusive smokers (1)** | **Dual users of NRT and tobacco (2)** | **Dual users of e-cigarettes and tobacco (3)** |  | **(3) vs. (1)** | **(3) vs. (2)** |  | **(3) vs. (1)** | **(3) vs. (2)** |
| Dual use for any reason, *n*^2^ | | 337 | 56 | 121 |  |  |  |  |  |  |
|  | Quit success | 35.3 (119) | 28.6 (16) | 31.4 (38) |  | 0.84 [0.54-1.31]  0.438 | 1.15 [0.57-2.29]  0.703 |  | 0.93 [0.57-1.51]  0.761 | 1.10 [0.52-2.33]  0.811 |
| ^1^ Adjusted for age, sex, ethnicity, social grade, number of cigarettes smoked per day, strength of urges to smoke, motivation to stop smoking, and past-year quit attempts at baseline, and year and quarter of survey. Models relating to the success of quit attempts also controlled for time since the quit attempt began and whether the quit attempt was abrupt or gradual, measured at 12-month follow-up.  ^2^ Number who reported at least one quit attempt in the past 12 months that started at least 1 month prior to the 12-month follow-up survey.  * *p*<0.05. | | | | | | | | | | |

| **Supplementary Table 3.** Sample characteristics at baseline according to use of e-cigarettes and NRT at 12-month follow-up among participants classified as exclusive smokers at baseline | | | | | |
| --- | --- | --- | --- | --- | --- |
|  | | **Use of neither e-cigarettes nor NRT**  **(*n*=916)** | **Users of NRT**  **(*n*=29)** | **Users of e-cigarettes**  **(*n*=140)** | ***p*^1^** |
| Age in years, % (*n*) | |  |  |  |  |
|  | 16-24 | 9.1 (83) | 6.9 (2) | 10.0 (14) | 0.084 |
|  | 25-34 | 9.6 (88) | 6.9 (2) | 16.4 (23) | - |
|  | 35-44 | 12.0 (110) | 10.3 (3) | 15.7 (22) | - |
|  | 45-54 | 19.4 (178) | 27.6 (8) | 22.9 (32) | - |
|  | 55-64 | 24.8 (227) | 17.2 (5) | 20.0 (28) | - |
|  | ≥65 | 25.1 (230) | 31.0 (9) | 15.0 (21) | - |
| Female sex, % (*n*) | | 47.9 (439) | 58.6 (17) | 47.1 (66) | 0.510 |
| White ethnicity, % (*n*) | | 94.2 (863) | 89.7 (26) | 92.9 (130) | 0.512 |
| Social grade C2DE, % (*n*) | | 51.2 (469) | 55.2 (16) | 52.1 (73) | 0.900 |
| Cigarettes per day, mean (SD) | | 12.26 (9.63) | 12.6 (6.03) | 13.68 (7.77) | 0.239 |
| Strength of urges (0-5), mean (SD) | | 1.91 (1.12) | 2.03 (0.87) | 1.96 (1.09) | 0.767 |
| High motivation to quit, % (*n*) | | 8.6 (79) | 17.2 (5) | 13.6 (19) | 0.063 |
| Attempted to quit in past 12 months, % (*n*) | | 18.0 (165) | 31.0 (9) | 27.1 (38) | 0.011 |
| Dual use for harm reduction, % (*n*) | | - | 51.7 (15) | 55.0 (77) | 0.747 |
| ^1^ *p* value for the association between each variable and group (dual use of e-cigarettes, dual use of NRT, and exclusive smoking). | | | | | |

| **Supplementary Table 4.** Cross-sectional associations at 12-month follow-up between use of e-cigarettes among past-year smokers and successful cessation (for at least one month) among those who made a quit attempt, adjusting for covariates measured at baseline | | | | | | | | | | |
| --- | --- | --- | --- | --- | --- | --- | --- | --- | --- | --- |
|  | | **Prevalence, % (*n*)** | | |  | **OR [95% CI]**  ***p*** | |  | **OR_adj_ [95% CI]^1^**  ***p*** | |
|  | | **Neither e-cigarettes nor NRT (1)** | **Use of NRT (2)** | **Use of e-cigarettes (3)** |  | **(3) vs. (1)** | **(3) vs. (2)** |  | **(3) vs. (1)** | **(3) vs. (2)** |
| Dual use for any reason, *n*^2^ | | 286 | 40 | 134 |  |  |  |  |  |  |
|  | Quit success | 29.4 (84) | 30.0 (12) | 39.6 (53) |  | 1.57 [1.02-2.42]  0.039 | 1.53 [0.71-3.26]  0.275 |  | 1.64 [1.03-2.60]  0.035 | 1.49 [0.66-3.36]  0.339 |
| ^1^ Adjusted for age, sex, ethnicity, social grade, number of cigarettes smoked per day, strength of urges to smoke, motivation to stop smoking, and past-year quit attempts at baseline, and year and quarter of survey. Models relating to the success of quit attempts also controlled for time since the quit attempt began and whether the quit attempt was abrupt or gradual, measured at 12-month follow-up.  ^2^ Number who reported at least one quit attempt in the past 12 months that started at least 1 month prior to the 12-month follow-up survey.  * *p*<0.05. | | | | | | | | | | |
